# Tracking Self-reported Symptoms and Medical Conditions on Social Media During the COVID-19 Pandemic: Infodemiological Study

**DOI:** 10.2196/29413

**Published:** 2021-09-28

**Authors:** Qinglan Ding, Daisy Massey, Chenxi Huang, Connor B Grady, Yuan Lu, Alina Cohen, Pini Matzner, Shiwani Mahajan, César Caraballo, Navin Kumar, Yuchen Xue, Rachel Dreyer, Brita Roy, Harlan M Krumholz

**Affiliations:** 1 Center for Outcomes Research and Evaluation Yale New Haven Hospital New Haven, CT United States; 2 College of Health and Human Sciences Purdue University West Lafayette, IN United States; 3 Department of Chronic Disease Epidemiology Yale School of Public Health New Haven, CT United States; 4 Section of Cardiovascular Medicine Department of Internal Medicine Yale School of Medicine New Haven, CT United States; 5 Skai Tel-Aviv Israel; 6 Department of Sociology Yale University New Haven, CT United States; 7 Institute for Network Science Yale University New Haven, CT United States; 8 Foundation for a Smoke-Free World New York, NY United States; 9 Department of Emergency Medicine Yale School of Medicine New Haven, CT United States; 10 Department of Medicine Yale School of Medicine New Haven, CT United States; 11 Department of Health Policy and Management Yale School of Public Health New Haven, CT United States

**Keywords:** health conditions, symptoms, mental health, social media, infoveillance, public health surveillance, COVID-19, pandemic, natural language processing

## Abstract

**Background:**

Harnessing health-related data posted on social media in real time can offer insights into how the pandemic impacts the mental health and general well-being of individuals and populations over time.

**Objective:**

This study aimed to obtain information on symptoms and medical conditions self-reported by non-Twitter social media users during the COVID-19 pandemic, to determine how discussion of these symptoms and medical conditions changed over time, and to identify correlations between frequency of the top 5 commonly mentioned symptoms post and daily COVID-19 statistics (new cases, new deaths, new active cases, and new recovered cases) in the United States.

**Methods:**

We used natural language processing (NLP) algorithms to identify symptom- and medical condition–related topics being discussed on social media between June 14 and December 13, 2020. The sample posts were geotagged by NetBase, a third-party data provider. We calculated the positive predictive value and sensitivity to validate the classification of posts. We also assessed the frequency of health-related discussions on social media over time during the study period, and used Pearson correlation coefficients to identify statistically significant correlations between the frequency of the 5 most commonly mentioned symptoms and fluctuation of daily US COVID-19 statistics.

**Results:**

Within a total of 9,807,813 posts (nearly 70% were sourced from the United States), we identified a discussion of 120 symptom-related topics and 1542 medical condition–related topics. Our classification of the health-related posts had a positive predictive value of over 80% and an average classification rate of 92% sensitivity. The 5 most commonly mentioned symptoms on social media during the study period were anxiety (in 201,303 posts or 12.2% of the total posts mentioning symptoms), generalized pain (189,673, 11.5%), weight loss (95,793, 5.8%), fatigue (91,252, 5.5%), and coughing (86,235, 5.2%). The 5 most discussed medical conditions were COVID-19 (in 5,420,276 posts or 66.4% of the total posts mentioning medical conditions), unspecified infectious disease (469,356, 5.8%), influenza (270,166, 3.3%), unspecified disorders of the central nervous system (253,407, 3.1%), and depression (151,752, 1.9%). Changes in posts in the frequency of anxiety, generalized pain, and weight loss were significant but negatively correlated with daily new COVID-19 cases in the United States (r=-0.49, r=-0.46, and r=-0.39, respectively; *P*<.05). Posts on the frequency of anxiety, generalized pain, weight loss, fatigue, and the changes in fatigue positively and significantly correlated with daily changes in both new deaths and new active cases in the United States (r ranged=0.39-0.48; *P*<.05).

**Conclusions:**

COVID-19 and symptoms of anxiety were the 2 most commonly discussed health-related topics on social media from June 14 to December 13, 2020. Real-time monitoring of social media posts on symptoms and medical conditions may help assess the population’s mental health status and enhance public health surveillance for infectious disease.

## Introduction

The COVID-19 pandemic continues to spread worldwide, with more than 229 million confirmed cases and 4,7028,286 deaths in 188 countries as of September 21, 2021 [1]. As individuals are being encouraged to telecommute and self-quarantine, social media usage has surged by over 40%, emerging as a powerful tool for facilitating communication and disseminating information in a timely manner [2,3]. The general public and health care professionals use social media platforms for health surveillance; to share their feelings, opinions, knowledge, and experiences in relation to the COVID-19 pandemic; and interact with others who share similar characteristics or interests [4-7]. A growing number of people also use social media to seek and share health information that might otherwise be “invisible” to clinicians and medical researchers (eg, self-diagnosis and self-treated symptoms with over-the-counter medications) [8-10]. Harnessing publicly available health-related data posted on social media in real time has the potential to offer insights into how the pandemic impacts the mental health and general well-being of individuals and populations over time [2].

Although prior studies have demonstrated that social media discussions can influence health-related beliefs and behaviors, more studies are needed to understand how social media plays a role during the pandemic [11,12]. Since the emergence of the COVID-19 pandemic, an estimated 41% of US adults have delayed or avoided urgent and routine medical care during the pandemic owing to concerns about COVID-19 [13]. Real-time information regarding self-reported general health status at a population level is lacking. Most literature in this area of research has been focused particularly on mental health or COVID-19 symptoms, with Twitter frequently being utilized as the sole data source [5,14-18]. There was limited information regarding health-related discussions from social media sites other than Twitter. Furthermore, the predictive value of posts on COVID-19 symptoms or related medical conditions on social media sites other than Twitter has not yet been ascertained [19,20]. Extracting and analyzing health-related data from multiple social media sources might provide novel ways of measuring the health status and the full spectrum of symptoms and illness of the population in real time [21,22].

As such, we created a dashboard to extract and monitor posts mentioning symptoms and medical conditions from social media sites other than Twitter over the course of the COVID-19 pandemic. In this study, we sought to answer the following questions: (1) what symptoms and medical conditions were people discussing on social media platforms other than Twitter during the COVID-19 pandemic? (2) How have discussions of symptoms and medical conditions on social media changed over a 6-month period during the pandemic? (3) Were daily fluctuations in health-related social media conversations associated with daily changes in COVID-19 statistics (new cases, new deaths, new active cases, and new recovered cases) in the United States?

## Methods

### Data Collection

We included English-language social networks and forums worldwide, such as Facebook public pages, Reddit, 4Chan, and the comments sections of news sites such as ABC News [23]. We defined forums as thread-based message boards and topic-specific pages [24]. We chose these sources to provide diversity because they have been studied less than Twitter in this area [25,26]. Additionally, the user base profile of our sources appeared to be more representative of the demographic profile of the broader US population than Twitter. While both Twitter and Reddit were popular among US adults aged ≤30 years, those who lived in urban areas, and were male [27], Facebook appeared to be more popular among female users and US adults older than 30 years [27].

Furthermore, even though there is an overlap between the affordance among our sources and Twitter, Reddit users have more anonymity as they do not need to register an account to access the majority of the content, thus allowing for greater participation [25]. Lastly, forums such as Reddit allow lengthy submissions and are usually topic-specific, which grant opportunities to cover the sensitive topics of our study (eg, mental health disorders and symptoms), which may not typically be discussed on social media [26,28]. The greater the length of the comments (eg, 40 words per comment for Reddit vs <15 words in tweets, on average) and less frequent use of hashtags associated with forums, which also makes it possible to apply more complex natural language processing (NLP) algorithms more accurately to classify sample posts [26].

We partnered with Signals Analytics, an advanced analytics company, to obtain access to target data sources from a third-party data vendor (NetBase) and to conduct the analysis [29,30]. In order to geotag posts, NetBase used a combination of geotagged social media messages, author profiles, and each country’s unique website domain suffix (eg, “.ca” for Canada). All the acquired data were then deidentified by NetBase and transferred to Signals Analytics for analysis.

We also gathered data on COVID-19 cases from the COVID-19 dashboard developed by the Center for System Science and Engineering at Johns Hopkins University, which provides the most comprehensive and up-to-date information on COVID-19 trends [1]. Using the RapidAPI application programming interface (API) [31], we updated the COVID-19 statistics (daily new cases or incidence) on a daily basis.

In this study, all personal identifying information such as usernames, emails, and IP addresses were removed before analysis. The study was exempt from institutional review board review at Yale University as it used publicly available, anonymized data.

### Data Analysis

For the analysis of data on symptoms and medical conditions being discussed on social media platforms between June 14 (when many countries began to lift major COVID-19 restrictions) and December 13, 2020 (when the first shipment of the COVID-19 vaccine arrived in the United States), we began by applying NLP algorithms to process social media posts collected from data sources during the study period, and then classified these posts in accordance with symptoms and medical conditions being mentioned.

To accomplish this, NetBase ran a daily scheduled data extraction query that we designed for the study on over 300 million web-based data sources (Multimedia Appendix 1). Additionally, we performed the following filtering steps to include posts relevant to our research questions. First, NLP algorithms were run, and advertisements and posts on sites for pornography were removed (Multimedia Appendix 1). Next, we applied a taxonomy of over 3000 health-related topics to identify key words, phrases, and statements mentioning symptoms and medical conditions (Multimedia Appendix 1). Social media posts that did not contain any of the taxonomy terms or symptoms and medical conditions as keywords were then deleted. Lastly, we removed redundant posts, blog posts, and news articles to ensure that the analysis was based on unique posts from social networks, forums, and comments only.

To evaluate the performance of the NLP algorithms and taxonomy classifications of symptoms and medical conditions, we applied the taxonomy to 4 sets of independent 100-post samples and calculated the positive predictive value and sensitivity of the classification (Multimedia Appendix 1). The algorithms used to identify symptoms and medical conditions topics in our study have been previously validated using real-world data to assess the public’s behaviors and perceptions toward COVID-19 [32]. Our study methodology has also been used to provide insights into the characterization and prediction of e-cigarette or vaping product use–associated lung injury outbreaks, known as the EVALI study [33].

Our taxonomy was organized into three levels: categories, subcategories, and topics. Symptoms and medical conditions were the 2 main categories in the taxonomy (Table 1 and Multimedia Appendix 1). The symptoms category included 98 non–COVID-19 topics (symptoms), which were grouped into 7 subcategories based on the affected organ or systems (eg, cardiovascular or respiratory systems). A list of 22 COVID-19–related topics (symptoms) was included as a separate symptom subcategory. The list of COVID-19–related symptoms was defined as outlined by the Centers for Disease Control and Prevention (CDC) on December 22, 2020 [34]. Because our algorithms captured all posts that mentioned any of the listed COVID-19 symptoms in the COVID-19–related symptom subcategory, the included posts may not necessarily represent discussions of symptoms experienced by patients with COVID-19. The medical condition category included 2200 topics (medical diagnoses), which were grouped into 10 subcategories. Categories, subcategories, and topics in the taxonomy were not mutually exclusive; each post could be assigned to multiple categories, subcategories, or topics.

We also created content filters to retain posts mentioning COVID-19 for further analysis. We applied 2 filters, COVID-19 disease status and COVID-19 diagnostic methods, to identify discussions on COVID-19 disease status (tested positive or negative, symptomatic or asymptomatic, recovered, and exposed to a confirmed patient) and diagnostic methods (COVID-19 testing, self-diagnosis, and remote diagnosis). These more restrictive searches were conducted by activating the 2 additional filters using the NLP algorithm, and the resulting posts from that search may not indicate the author’s COVID-19 status.

To explore how the discussion of symptoms and medical conditions on social media changed from June 14 to December 13, 2020, we determined the number of posts that included a discussion of each symptom and medical condition over time using NLP classification (Multimedia Appendix 1). To assess whether the frequency of symptom posts was associated with daily COVID-19 statistics, we performed Pearson correlation analysis to determine correlations among the top 5 most discussed symptoms and daily COVID-19 statistics (new cases, new deaths, new active cases [total cases minus recovered and those who have died], and new recovered cases). Additionally, we calculated Pearson correlation coefficients between frequency changes in each of the 5 symptoms and daily fluctuation in any COVID-19 statistic. A 2-tailed *P* value of <.05 was used to indicate statistical significance. Both posts and COVID-19 statistics used in these analyses were restricted to the United States.

Additionally, we compared the trends of the 5 most frequently mentioned symptoms and medical conditions from June 14 to August 31, 2021 (when the United States crossed the 6 million COVID-19 cases mark), to the trends observed from September 1 to December 13, 2020, by measuring the percent change between the 2 time periods in the number of posts including a discussion of each topic. We compared the 2 time periods to reveal changes in health-related conversations on social media at different stages of the pandemic, as prior literature focused primarily on the early stage of the pandemic (before June 2020). Our approach was also designed to contribute to a better understanding of the impact of COVID-19 on the public’s perceptions and attitudes toward different symptoms, medical conditions, and health care–seeking behaviors.

## Results

After social media posts were collected from sources, preprocessed, and classified in accordance with the taxonomy by NLP algorithms, our final sample included a total of 9,807,813 posts between June 14 and December 13, 2020, which mentioned at least 1 of the 120 symptoms or 1542 medical condition topics in our taxonomy (Table 1). Our taxonomy classification in the independent sample of 100 posts resulted in a positive predictive value of over 80% and an average classification rate of 92% sensitivity. Furthermore, based on indirect geotagging information provided by NetBase, approximately 70% of all posts collected by the search query were from the United States. The most prevalent symptom subcategory was “neuropsychological symptoms” (568,662/ 1,649,547, 34.5%), followed by the COVID-19–related symptoms subcategory (501,178/1,649,547, 30.4%). The most prevalent medical condition subcategory was “infectious disease” (6,052,068/8,158,266, 74.2%), followed by the subcategory of “psychiatric or mental health disorders” (484,505/8,158,266, 6.0%) (Table 1).

Irrespective of subcategories classification, the 5 most commonly mentioned symptom topics were anxiety (201,303, 12.20%, of the total posts mentioning symptoms), generalized pain (189,673, 11.5%), weight loss (95,793, 5.8%), fatigue (91,252, 5.5%), and coughing (86,235, 5.2%), accounting for 40.2% of all symptom posts combined (Table 2 and Multimedia Appendix 1). The 5 most discussed medical condition topics were COVID-19 (5,420,276, 66.4%, of the total posts mentioning medical conditions), unspecified infectious disease (469,356, 5.8%), influenza (270,166, 3.3%), unspecified disorders of the central nervous system (CNS) (253,407, 3.1%), and depression (151,752, 1.9%), and together they accounted for 80.5% of all medical conditions discussed on social media during the study period (Table 2 and Multimedia Appendix 1).

**Table 1:** Number of posts on symptoms and medical conditions mentioned on social media platforms by taxonomy topic (June 14 to December 13, 2020; N=9,807,813).

Relevant taxonomy categories and subcategories (number of topics)		Number of posts with symptoms or medical conditions	Percentage of all posts on symptoms or all medical conditions (%)
**Symptoms (n=1,649,547)**			
	Neuropsychological symptoms (17)	568,662	34.47
	COVID-19–related symptoms^a^ (22)	501,178	30.38
	Respiratory symptoms (7)	128,134	7.77
	Gastrointestinal symptoms (13)	120,621	7.31
	Dermal symptoms (16)	99,453	6.03
	Cardiovascular disease symptoms (4)	34,014	2.06
	Musculoskeletal symptoms (7)	33,604	2.04
	Other symptoms (34)	163,881	9.93
**Medical conditions (n=8,158,266)**			
	Infectious disease (80)	6,052,068	74.18
	Psychiatric or mental health disorders (21)	484,505	5.94
	Neurovascular and cardiovascular diseases (63)	465,675	5.71
	Respiratory disorders (17)	165,404	2.03
	Hematological and oncological disorders (127)	164,159	2.01
	Other disorders (1234)	828,786	10.13

^a^COVID-19–related symptoms were based on symptoms of COVID-19 (n=22) updated by the Centers for Disease Control and Prevention on December 22, 2020, which were as follows: runny nose, change in sense of taste, change in sense of smell, chills, bluish lips/face, inability to stay awake, fatigue, headache, sore throat, abdominal pain, vomiting, muscle pain/spasms, drowsiness, nausea, body aches, chest pain, itching/swelling, fever, confusion state, diarrhea, coughing, and difficulty breathing.

**Table 2:** Frequency of the top 5 most discussed symptoms and medical conditions on social media by taxonomy topic (June 14 to December 13, 2020; N=9,807,813).

Relevant taxonomy categories and topics		Number of posts with topics related to symptoms or medical conditions	Percentage of posts on all topics related to symptoms or all medical conditions (%)
**Symptoms (n=1,649,547)**			
	Anxiety	201,303	12.20
	Generalized pain	189,673	11.49
	Weight loss	95,793	5.81
	Fatigue	91,252	5.53
	Coughing	86,235	5.23
**Medical conditions (n=8,158,266)**			
	COVID-19	5,420,276	66.44
	Unspecified infectious disease	469,356	5.75
	Influenza	270,166	3.31
	Unspecified CNS^a^ disorders	253,407	3.11
	Depression	151,752	1.86

^a^CNS: central nervous system.

Within the COVID-19–related symptoms subcategory, fatigue (91,208, 32.9%) and coughing (86,222, 31.1%) were the most discussed COVID-19–related symptom topics (Table 3). Bluish lips/face (1019, 0.4%) and inability to stay awake (486, 0.2%) were the least commonly discussed COVID-19 symptoms.

After applying the COVID-19 disease status filter to all posts mentioning the top 5 most frequently mentioned symptoms and medical conditions, we noticed that within the posts classified with the medical condition of COVID-19, 62.9% had also discussed testing positive, and 9.1% of the discussions were related to asymptomatic COVID-19 (Table S2, Multimedia Appendix 1). Applying the COVID-19 diagnostic method filter revealed that the most popular COVID-19 diagnostic methods discussed were COVID-19 tests regardless of the symptom or medical condition subcategory (Table S2, Multimedia Appendix 1).

**Figure 1:**
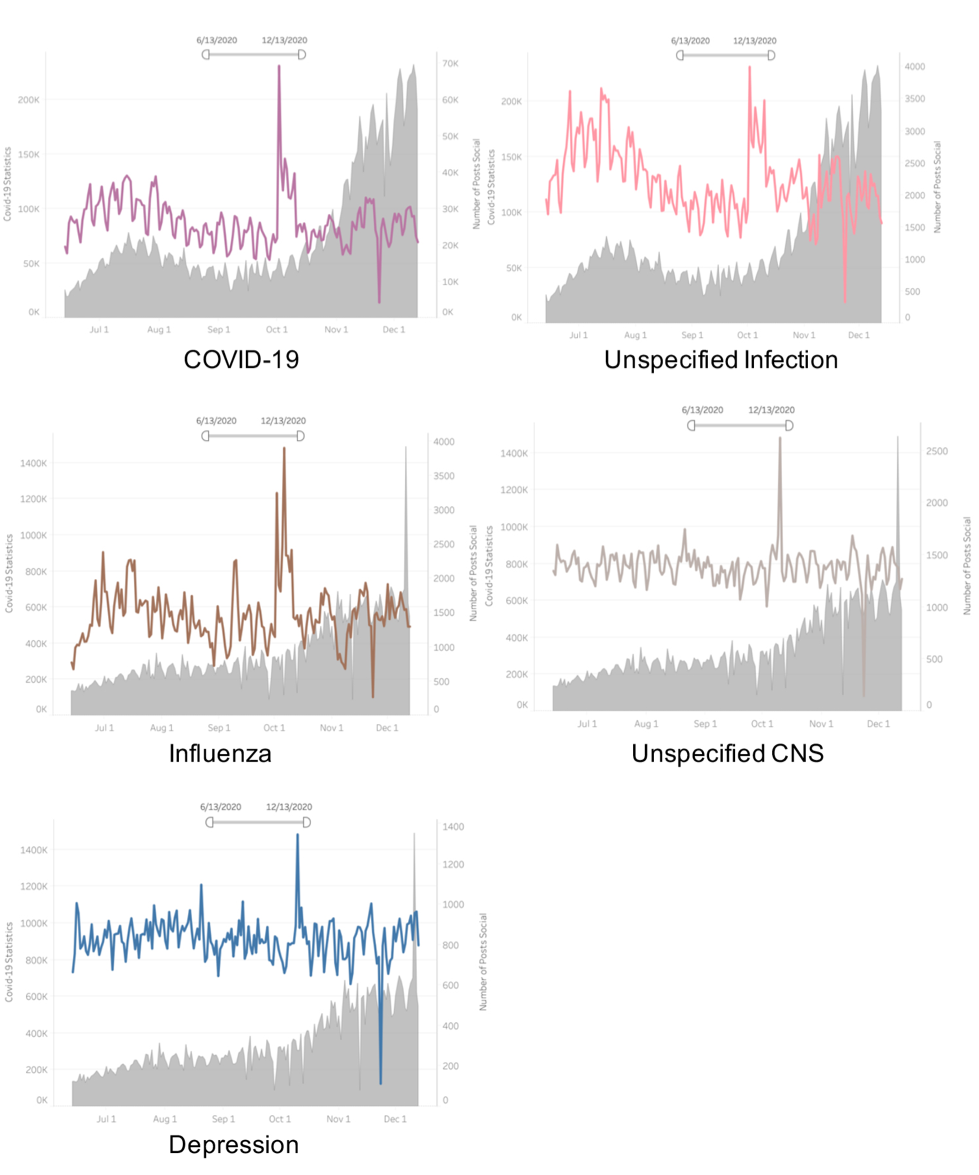
Associations between changes in new daily COVID-19 cases in the United States and the number of medical condition–related posts (June 13-December 13, 2020). (Note: the gray shaded area indicates daily active COVID-19 cases in the United States, while the colored curves showed fluctuations in posts mentioning different medical disorders during the study period). CNS: central nervous system.

**Figure 2:**
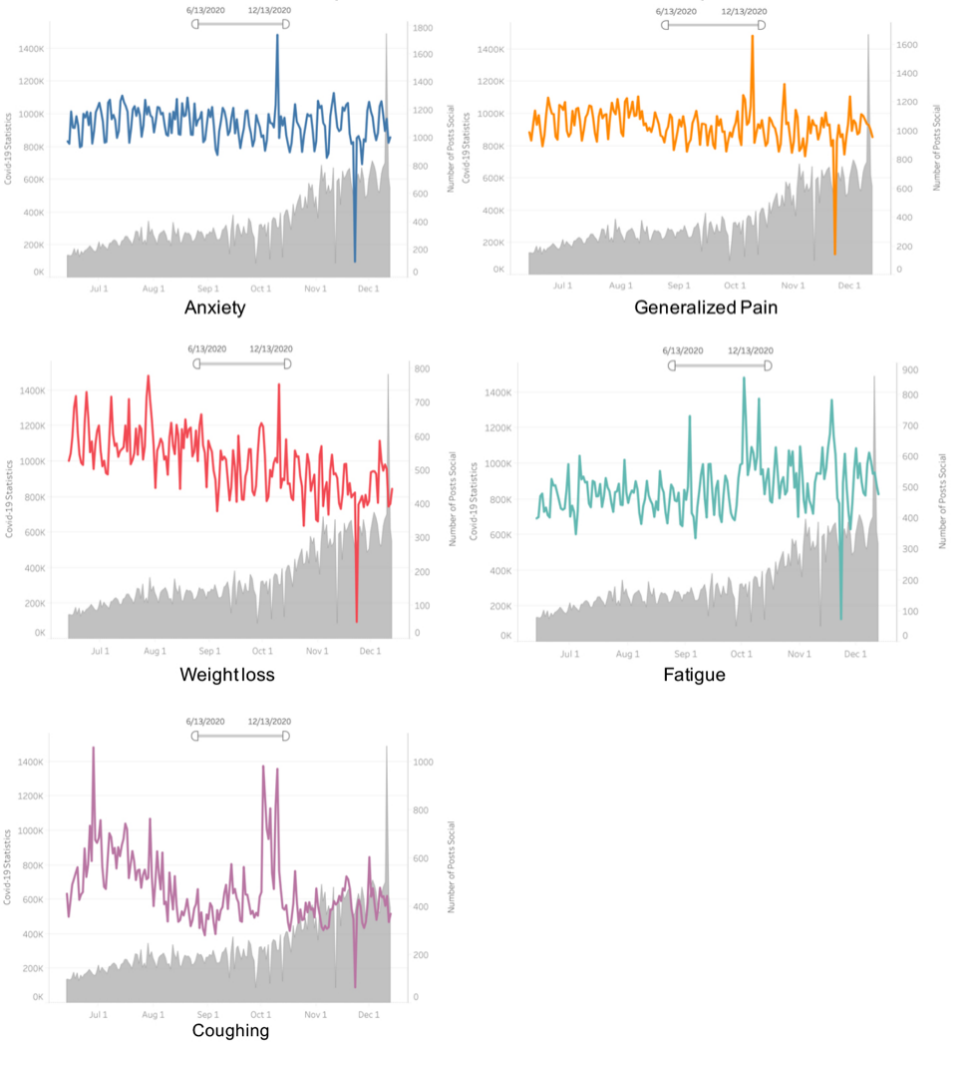
Associations between changes in new daily COVID-19 cases in the United States and the number of symptoms posts (June 13-December 13, 2020). (Note: the gray shaded area indicated daily active COVID-19 cases in the United States, while the colored curves showed fluctuations in posts mentioning different symptoms during the study period).

The pattern of changes in top 5 commonly mentioned posts of medical conditions or symptoms and the fluctuation of daily new COVID-19 cases in the United States were displayed in Figures 1 and 2. We noticed a significant increase in daily frequency of posts mentioning the top 5 symptom- and medical condition–related topics in October 2020 and a decrease in late November-December 2020 (Multimedia Appendix 1). Statistical analysis showed that the frequency of symptom posts that was strongly associated with daily new cases included changes in anxiety (r=–0.49; *P*=.009), changes in generalized pain (r=–0.46; *P*=.01), and changes in weight loss (r=–0.39; *P*=.04) (Multimedia Appendix 1). The frequency of symptom-related posts that strongly correlated with daily changes in both new deaths and new active cases included anxiety (r=0.49, *P*=.008; r=0.59, *P*=.002, respectively); generalized pain (r=0.48, *P*=.01; r=0.59, *P*=.001, respectively); weight loss (r=0.39, *P*=.04; r=0.48, *P*=.01, respectively); fatigue (r=0.48, *P*=.01; r=0.53, *P*=.049; and changes in fatigue (r=0.09, *P*=.001; r=0.48, *P*=.009, respectively) (Multimedia Appendix 1).

Correlations between the frequency of the 4 most commonly discussed symptoms and daily recovered cases were significant, and their Pearson correlation coefficients were –0.43 for anxiety, –0.44 for generalized pain, –0.55 for weight loss, and –0.51 for coughing, which indicated a negative and moderate correlation among them (Multimedia Appendix 1).

When examining changes in the frequency of the top 5 most commonly mentioned symptom topic discussions over the 6-month study period, we noted a 24% increase in symptom posts mentioning anxiety, generalized pain, and fatigue during September 1-December 13, 2020 (vs June 14-August 31, 2020) (Multimedia Appendix 1). Compared to June 14-August 31, 2020, posts mentioning the medical condition–related topics influenza, unspecified CNS disorders, and depression increased by more than 27% during September 1-December 13, 2020 (Multimedia Appendix 1). In terms of changes within the COVID-19–related symptoms subcategory, social media posts mentioning runny nose and change in the sense of taste and smell increased over 64%, while posts mentioning difficulty breathing decreased 1.5% during September 1-December 13, 2020 (vs June 14-August 31, 2020) (Multimedia Appendix 1).

**Table 3:** Comparing changes in the number of posts on COVID-19 symptoms between June 14 and August 31, 2020, with those in September 1 to December 13, 2020 (N=277,401).

COVID-19–related symptoms per the Centers for Disease Control and Prevention’s definition^a^	Posts mentioning this COVID-19 symptoms, n (%)	Posts during June 14-August 31, 2020, n	Posts during September 1-December 13, 2020, n	Changes in the number of posts, %
Fatigue	91,208 (32.88)	36,876	54,332	47.33
Coughing	86,222 (31.08)	41,163	45,059	9.46
Fever	59,906 (21.59)	27,729	32,177	16.04
Headache	41,693 (15.02)	18,052	23,641	30.96
Vomiting	39,103 (14.09)	17,364	21,739	25.19
Difficulty breathing	33,589 (12.11)	16,917	16,672	Decreased 1.45
Nausea	29,103 (10.49)	13,039	16,064	23.19
Itching/swelling	28,337 (10.22)	12,953	15,384	18.77
Sore throat	14,694 (5.29)	6424	8270	28.74
Diarrhea	14,140 (5.09)	6716	7424	10.54
Chest pain	9412 (3.39)	4255	5157	21.19
Abdominal pain	9238 (3.33)	4080	5158	26.42
Runny nose	8283 (2.98)	3029	5254	73.46
Body aches	7871 (2.84)	3540	4331	22.34
Change in sense of taste	6510 (2.35)	2447	4063	66.04
Muscle pain/spasms	6321 (2.28)	2816	3505	24.47
Change in sense of smell	6192 (2.23)	2340	3852	64.62
Confusional state	3716 (1.34)	1737	1979	13.93
Chills	2879 (1.04)	1141	1738	52.32
Drowsiness	1256 (0.45)	560	696	24.29
Bluish lips/face	1019 (0.37)	404	615	52.23
Inability to stay awake	486 (0.18)	195	291	49.23

^a^The list of COVID-19 symptoms was updated on December 22, 2020, in accordance with the Centers for Disease Control and Prevention’s update. Our algorithms captured all posts mentioning any of these symptoms in the COVID-19 symptom subcategory; consequently, the posts may not necessarily represent patients discussing their own COVID-19 symptoms.

## Discussion

### Principal Findings

In this study, we collected and analyzed web-based posts from forums and comments on news sites between June 14 and December 13, 2020. We found that a wide variety of symptoms and medical conditions topics were discussed on non-Twitter social media. While the vast majority of discussions were about COVID-19 infection and COVID-19–related symptoms (as defined by the CDC), neuropsychological symptoms (eg, anxiety) and other medical conditions (eg, infectious diseases and psychiatric disorders) were also frequently mentioned. Additionally, we noticed that changes in posts frequency of anxiety, generalized pain, and weight loss were significant but negatively correlated with daily new COVID-19 cases in the United States, and that the frequency of posts on anxiety, generalized pain, weight loss, fatigue, and the changes in fatigue positively and significantly correlated with daily changes in both new deaths and new active cases in the United States. As COVID-19 cases continued to rise globally, the cumulative volume of posts mentioning anxiety, generalized pain, fatigue, influenza, unspecified CNS disorders, and depression increased from September 1 to December 13, 2020 (compared to June 13 to August 31, 2020).

Our findings expand on previous observations regarding the mental health effects of the COVID-19 pandemic among social media users by presenting a more complete picture of health-related topics discussed on social media [18]. Our results not only confirm the findings from previous studies that showed high levels of anxiety and depression mentioned by social media users during the pandemic [35,36] but also revealed that the frequency of anxiety and other general health symptom–related posts, including generalized pain, weight loss, and fatigue, was significantly correlated with daily COVID-19 statistics. These data support the idea that social media represents a potential powerful source of information for health care professionals to draw real-time estimations about population health status [18,21]. Understanding health symptom posts commonly associated with COVID-19 statistics may inform public health researchers, clinicians, and policymakers to take timely and appropriate public health and clinical measures accordingly.

Further, as access to the internet becomes more widely available and with the anonymity of social media, people who face barriers to accessing health care and those who have mental health symptoms may use social media to speak openly about their health experiences and seek help [21,37]. Collectively, these results further justify our approach to monitoring symptoms and medical condition posts on social media during the pandemic, and call for further investigation of the possibility of using social media analytics to gain insights into the population’s symptoms, including mental health symptoms, which are difficult to monitor outside of the health system, health threats, and to enhance public health preparedness.

As the pandemic progresses, obtaining information on the symptom profile of COVID-19 could help to better diagnose and treat the disease. There has been increasing recognition of the importance of extracting social media information to explore symptom experience and disease progression among patients with COVID-19 [38]. Although we did not restrict our analysis to only social media posts mentioning COVID-19 and could not verify the authors’ disease status, the most discussed COVID-19–related symptoms we found (eg, fatigue, cough, fever, headache, and difficulty breathing) were among the most common symptoms reported by patients with COVID-19 in other studies [39-41]. Based on information extracted by applying COVID-19 disease status and diagnostic methods filters, we found that nearly 40% of non-Twitter social media users who discussed the top 5 most commonly mentioned symptom topics, such as fatigue and cough, also talked about the topic of having tested positive for COVID-19.

We also noticed that approximately 15% of these discussions were related to asymptomatic COVID-19. While an in-depth exploration of these posts using qualitative analysis or sentiment analysis is necessary to help verify the users’ COVID-19 disease status, our preliminary data indicate the potential for extracting information from social media to understand the full spectrum of symptoms experienced by patients with COVID-19. Interestingly, we noticed an increase of over 60% in the volume of posts mentioning less common COVID-19 symptoms such as changes in the senses of taste and smell during the second stage of our study period (September 1 to December 13, 2020). This surge may be partly due to improvements in knowledge and awareness of COVID-19 symptoms in the general population as the 2 symptoms were recently added to the COVID-19 symptom lists of the CDC and the World Health Organization (late April 2020 and early May 2020, respectively).

While there have been fluctuations in the volume of social media posts on a day-to-day basis, there appeared to be seasonal variation in the volume of discussion of symptoms and medical conditions. We noticed that the volume of most health-related discussions increased more from September 1 to December 13, 2020, than from June 14 to August 31, 2020. These changes may have been due to a combination of colder weather in the northern hemisphere and social distancing and limitations on daily life during the pandemic as well as the second wave of COVID-19, resulting in more social media users and more people being restricted indoors [42]. Additionally, there were several inflection points in the volume of discussion of symptoms and medical conditions in the last 6 months. These changes appeared to have coincided with major news stories and national events, echoing findings from other studies that showed the potential impact of media coverage on web-based discussions [6,18]. For example, the volume of all 5 commonly mentioned symptoms (anxiety, generalized pain, weight loss, fatigue, and cough) and 2 medical conditions (unspecified CNS and depression) peaked on October 10, 2020, the day on which hurricane Delta struck Louisiana and nearby states and left 730,000 homes and businesses without power [43]. However, our study did not find evidence of an association between changes in the volume of symptom discussion over time and the trend of daily new confirmed cases of COVID-19 in the United States.

### Limitations

Our study has several limitations. First, information on geolocation, demographics, and COVID-19 disease status was not available for all social media users in the study, owing to various legal limitations (such as General Data Protection Regulation of the European Union). This might have introduced a sampling bias if there were significant differences between social media users’ characteristics in our project and the real world. However, by collaborating with social media analytics companies, we have maximized our ability to access thousands of social media data sources worldwide, thus minimizing the possibility of sampling bias. Additionally, the majority of social media users in our study were from the United States. The findings, therefore, may not be generalizable in their application to users located in other countries. Further, we did not conduct formal statistical analyses beyond comparing the trends differences in frequency of health-related posts and new COVID-19 cases; hence, further testing is needed to confirm the associations between patterns of changes in symptom/medical condition posts and the fluctuations of COVID-19 statistics over time. Finally, we did not perform sentiment analysis or qualitative analysis in the study and did not verify whether authors who discussed COVID-19–related topics had COVID-19 themselves. We hope to accomplish and report this analysis in a future study. We also hope that other studies on social media’s role in public health will replicate and validate our exploratory findings in non-Twitter social media platforms.

### Conclusions

In this study, we classified web-based posts collected from June 14 to December 13, 2020, in accordance with discussions of symptoms and medical conditions. Neuropsychological symptoms such as anxiety were the most frequently mentioned symptom subcategory. Furthermore, COVID-19 infection was the most commonly mentioned medical condition. Our analysis also showed that frequency of anxiety and other general health symptoms posts, including generalized pain, weight loss, and fatigue, was significantly correlated with daily COVID-19 statistics in the United States. Additionally, health-related discussions were greater from September 1 to December 13, 2020, than from June 14 to August 31, 2020, aligning with the increase in COVID-19 cases in the United States during the winter months. These preliminary findings show promise for real-time monitoring of social media posts to measure the mental health status of a population during a global public health crisis and to assess the public’s main health needs that have not been captured or met by the existing health system. Future research may incorporate information from social media into predictive models for the detection of emerging infectious diseases.

## Appendix

Multimedia Appendix 1 Supplementary methods, figures, and tables.

## Abbreviations

API: application programming interface
CDC: Centers for Disease Control and Prevention
CNS: central nervous system
EVALI: e-cigarette or vaping use-associated lung injury
NLP: natural language processing
WHO: World Health Organization
