# Supplementary material for: Tracking Self-reported Symptoms and Medical Conditions on Social Media During the COVID-19 Pandemic: Infodemiological Study
Source: JMIR Public Health Surveill. 2021 Sep 28;7(9):e29413. doi: 10.2196/29413 (PMC8480398; doi:10.2196/29413)
Supplement: Multimedia Appendix 1 [file publichealth_v7i9e29413_app1.docx]

# Tracking self-reported symptoms and medical conditions on social media during the COVID-19 pandemic

## Multimedia Appendix 1

### List of contents

**Method S1.** Developing data extraction query.

**Method S2.** Classification technologies, inclusions and exclusions of posts.

**Method S3**. Developing the taxonomy.

**Method S4.**Validating the taxonomy to demonstrate its utility.

**Method S5.** Applying the taxonomy (NLP algorithms) to extract posts mentioning symptoms and medical conditions topics.

**Figure S1a**. “RBG^a^” classified mentions in social media posts (September-October 2020).

**Figure S1b**. “George Floyd” classified mentions in social media posts (June-July 2020).

**Figure S2:** The 5 most commonly mentioned symptoms on social media during the period of June 14 to December 13, 2020.

**Figure S3:** The 5 most commonly mentioned medical disorders on social media during the period of June 14 to December 13, 2020.

**Figure S4.** Comparing changes in the 5 most commonly mentioned symptoms on social media between June 13-Aug 31, 2020, to September 1-December 13, 2020.

**Figure S5.** Comparing changes in the 5 most commonly mentioned medical disorders on social media between June 13-Aug 31, 2020 to September 1-December 13, 2020.

**Figure S6.** Comparing changes in the 10 most commonly mentioned COVID-19 symptoms between June 13-Aug 31, 2020 and September 1-December 13, 2020.

**Table S1**. Example of original posts classified as taxonomy topics by NLP algorithms.

**Table S2**. Frequency of COVID-19 disease status and diagnostic methods discussions for the 5 most commonly mentioned symptoms and medical conditions on social media (June 14 to December 13, 2020, not indicative of a person’s COVID-19 status).

**Table S3**. Peak and trough dates of the number of social media posts for the 5 most commonly mentioned symptoms and medical conditions (June 14 and December 13, 2020).

**Table S4.** Daily changes in frequency of top 5 symptom posts associated with U.S. daily COVID-19 statistics during June 14 - December 13, 2020.

**Table S5.** Frequency of top 5 symptom posts associated with changes in U.S. daily COVID-19 statistics during June 14 - December 13, 2020.

**Table S6.** Daily changes in frequency of top 5 symptom posts associated with changes in U.S. daily COVID-19 statistics during June 14 - December 13, 2020.

**Table S7.** Frequency of top 5 symptom posts associated with U.S. daily COVID-19 statistics during June 14 - December 13, 2020.

**Method S1.** Developing data extraction query.

To capture all relevant social media posts mentioning symptoms and medical conditions during the study period, we initially extracted social media data using a broad query based on the larger project's scope and aims. Multiple analysts with different backgrounds and in-depth knowledge of our research topic constructed an initial list of health-related topics using symptoms and medical condition categories and subcategories relevant to our study's scope. For example, neuropsychological symptoms were considered as one of the symptom subcategories and anxiety was a topic within the neuropsychological symptom subcategory; infectious disease was a medical condition subcategory, and COVID-19 was one of the topics within the infectious disease subcategory. The analysts studied the posts from various social media data sources mentioning symptom and medical conditions categories and subcategories, and were able to extend the initial list of health-related topics. The comprehensive list also contained contextual data and major national or global events, the days of the week, and U.S. specific events such as "Ruth Bader Ginsburg" and "George Floyd".

Based on the list of symptoms and medical condition topics identified, NetBase ran a contextually-based proprietary query, which approach was also based on Boolean combinations of health-related topics and the relationships between them. In general, the query was built from over 200 lines, each containing either a value (or values combinations), or an operator. The initial query was broad and based on Boolean combination of several terms in various categories (such as “symptoms”, “medical conditions”, “health behavior”, etc.). There were many unique terms within each category/subcategory, and the use of Boolean terms helped create thousands of potential search combinations.

Due to our study purpose of gaining insights into how the pandemic impacts the mental and general well-being of the public, the comprehensive lists of topics (e.g., national events) that were included as part of the query were not validated in the context of COVID-19. Thus, we can’t guarantee that those who had discussed a certain symptom or disease condition also had COVID-19 or that they were discussing such topic in the context of COVID. Such approach allows our study to uncover a broader range of health-related topics that were being discussed among social media users with and without COVID-19 during the pandemic.

**Method S2.** Classification technologies, inclusions and exclusions of posts.

Our classification was based on a combination of machine learning (ML) and human-consensus based binary classification (inclusion, exclusion). While some posts were classified based on the existence of a specific keyword or keyword combinations, others were classified based on the presence of adjacent values to the term that was searched for. No one specific method was used per each of the values that we classified in our study. Approximately 500-600 posts were reviewed by a group of 3-4 Signals analysts during the classification process. As a first step, the analysts went over the posts and found potential word combinations and values to exclude irrelevant data. After that, they went over this process again to find what might have been missed during the first step. Usually, such process required 3-5 cycles until the amount of unwanted data became negligible. Whenever the analysts found something was missed, it was added to the initial exclusion rule-bases.

In terms of agreement between annotators, Signals’ analysts were trained to determine on their own whether a certain rule fits or not to a specific situation. In case of uncertainty, the 3-4 analysts discuss internally, and upon need, a team leader can make a final decision.

**Method S3**. Developing the taxonomy.

We created the taxonomy of health-related topics to classify symptoms and medical conditions posts. This taxonomy was created based on: 1) A literature review of symptoms and medical conditions commonly mentioned in journals, disease and health problems classification databases, and social media platforms during the COVID-19 pandemic; 2) Consultation with clinical experts; 3) Alignment with our research objectives. Sample disease and health problems classification databases used to create our taxonomy included the 10^th^ revision of the International Statistical Classification of Disease and Related Health Problems (ICD-10) by the World Health Organization, Anatomical Therapeutic Chemical (ATC) Classification by the World Health Organization, and National Organization for Rare Disorders (NORD). Study researchers conducted the literature review on June 18, 2020, and used the PubMed, medRxiv, and Google Scholar databases. In addition, the literature review included scholarly articles from medicine, nursing, public health, psychology, and sociology to identify health-related topics. A total of 100 articles were reviewed during the literature review. We chose not to continue the literature review after reviewing 100 articles as more data revealed no unrecognized theme and our reviewers agreed saturation had been reached. The taxonomy was examined by clinical experts (our team members: S.M. & C.C.). They added additional terms that they both agreed were relevant to the study to the taxonomy and finalized it based on our research objectives.

We also used a process with multiple steps to further develop and validate the keywords list and rules corresponding to each topic entered into the taxonomy. Our analysts used online dictionaries, medical dictionaries, and thesauruses to obtain keywords for each topic in our taxonomy. After creating a preliminary keywords list, we took multiple random data samples for manual examination to identify other keywords, frequent typographical errors, slang, and medical term context to create greater accuracy. We also used the context of terms to develop inclusion and exclusion criteria for keywords.

**Method S4.**Validating the taxonomy to demonstrate its utility.

To validate the taxonomy in our study, we used a total of 400 sample posts to demonstrate its utility. First, our analysts used a 100-post sample to calculate positive predictive value. After that, two analysts manually classified 3 separate 100-post samples to calculate sensitivity, focusing on 3 randomly chosen taxonomy values (300 posts in total). When there was disagreement, a third analyst reviewed the post, and the three analysts reached a consensus. The results of the manual classification of these 300 posts were later compared with the result after the taxonomy was applied using the NLP algorithms.

The algorithm had an over 80% positive predictive value for classifying symptoms and medical conditions for this independent dataset. The positive predictive value was calculated by first determining the number of posts correctly classified by the NLP algorithms to contain at least one of the taxonomy topics. These were placed in a ratio to all posts classified by the NLP algorithms to contain at least one of the taxonomy topics. Additionally, the Signals Analytics team conducted an ongoing quality assessment that included regular checks of taxonomy topics for accuracy, timely updates of the list of keywords and rules relevant to the changing nature of the social media data. Sensitivity analysis was performed by multiple analysts from a random sample of 3 separate taxonomy topics from which the analysts manually categorized 100 random posts. We checked these findings after taxonomy was applied to the posts. In our study, sensitivity was defined as the number of correct classifications of a symptom or medical condition topic using the NLP algorithms divided by the total number of social posts for the symptom or medical condition topic identified by our manual screening. The average classification rate was 92 % sensitivity after this taxonomy process.

Additionally, we validated our methodology by applying the algorithm to detect major news events that occurred during the study period. We were able to observe a dramatic increase in the volume of online discussion on topics relating to the event immediately following the occurrence (Figure S1a & S1b).

**Method S5.** Applying the taxonomy (NLP algorithms) to extract posts mentioning symptoms and medical conditions topics automatically.

Signals Analytics’ proprietary NLP algorithms and engines categorized the relevant health-related topics mentioned in the taxonomy. This "rulebase" technology depends on a RegEx curated rule set and language processing techniques which select keywords from free text. These rules also included synonyms, abbreviations, and related keywords for each of the taxonomy topics. These rules were then grouped together to ensure complete coverage for a specific symptom or medical condition topic. The rules were used as follows and as described below: 1. to identify the exact match in the text; 2. to determine the near-exact match in the text; 3. to correlate the match using stemming; 4. to use proximity operators to define the distance between two or more keywords; 5. and to pinpoint keywords to be included (positive classification) and excluded (negative classification, which overrode the original category).

Additionally, we excluded duplicates of posts (e.g., posts of the same date, title and content) using the proprietary NLP algorithms. If social media was posted more than once, it was considered unique to allow for counting the number of individuals discussing a particular topic.

To further stratify the posts, two filters were applied to all posts in our sample, such as applying a “COVID-19 disease status” filter to post mentioning symptom topic of anxiety. “COVID-19 diagnostic methods” was another filter among the posts mentioning positive test, as an illustration. All posts that discussed a taxonomy topic had filters applied and were applied by the NLP algorithms used to search for keywords to identify the filter topic discussed. For example, a filter of COVID-19 disease status applied to anxiety posts could determine the number of posts discussing both anxiety and COVID-19 disease status.

**Figure S1a**. “RBG^a^” classified mentions in social media posts (September-October 2020).

**
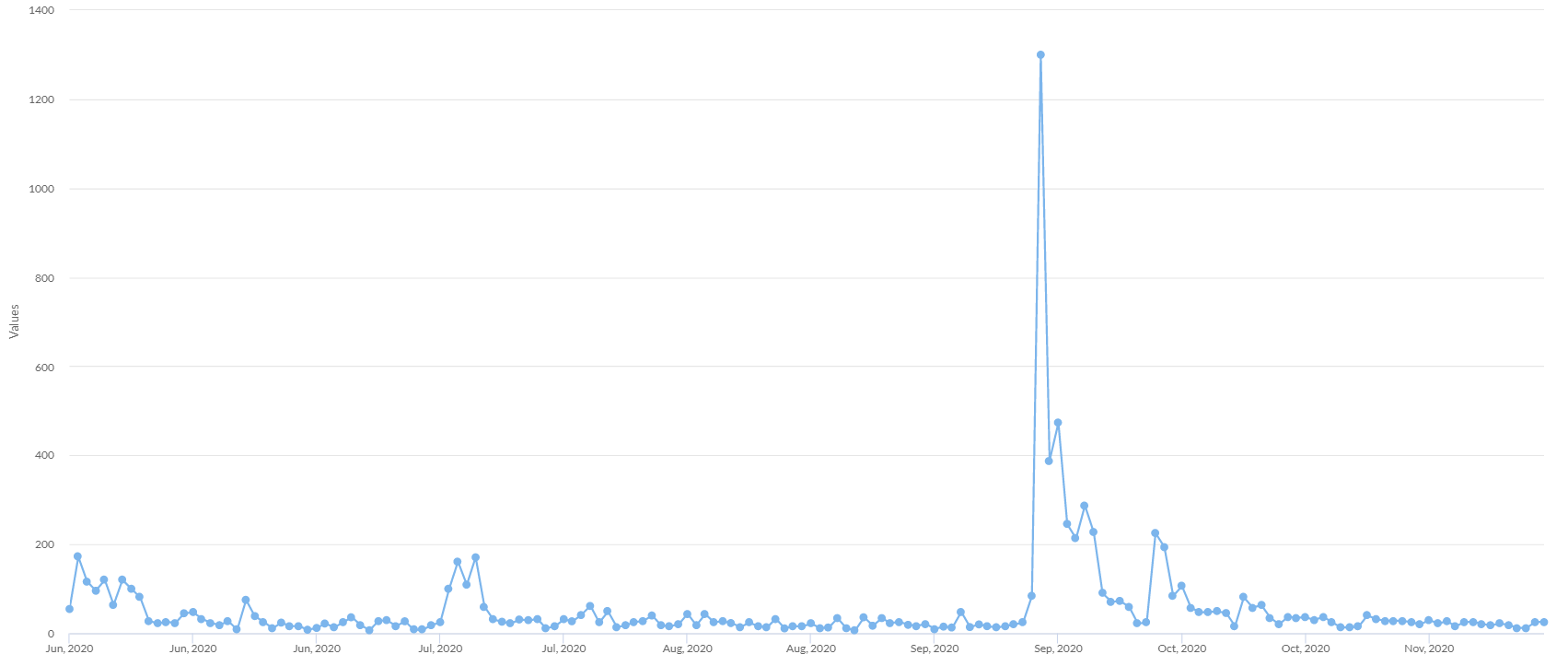
**

Number of Posts Including Topic Mentions

Time (Biweekly)

Abbreviations: a, Ruth Bader Ginsburg

**Figure S1b**. “George Floyd” classified mentions in social media posts (June-July 2020).

**
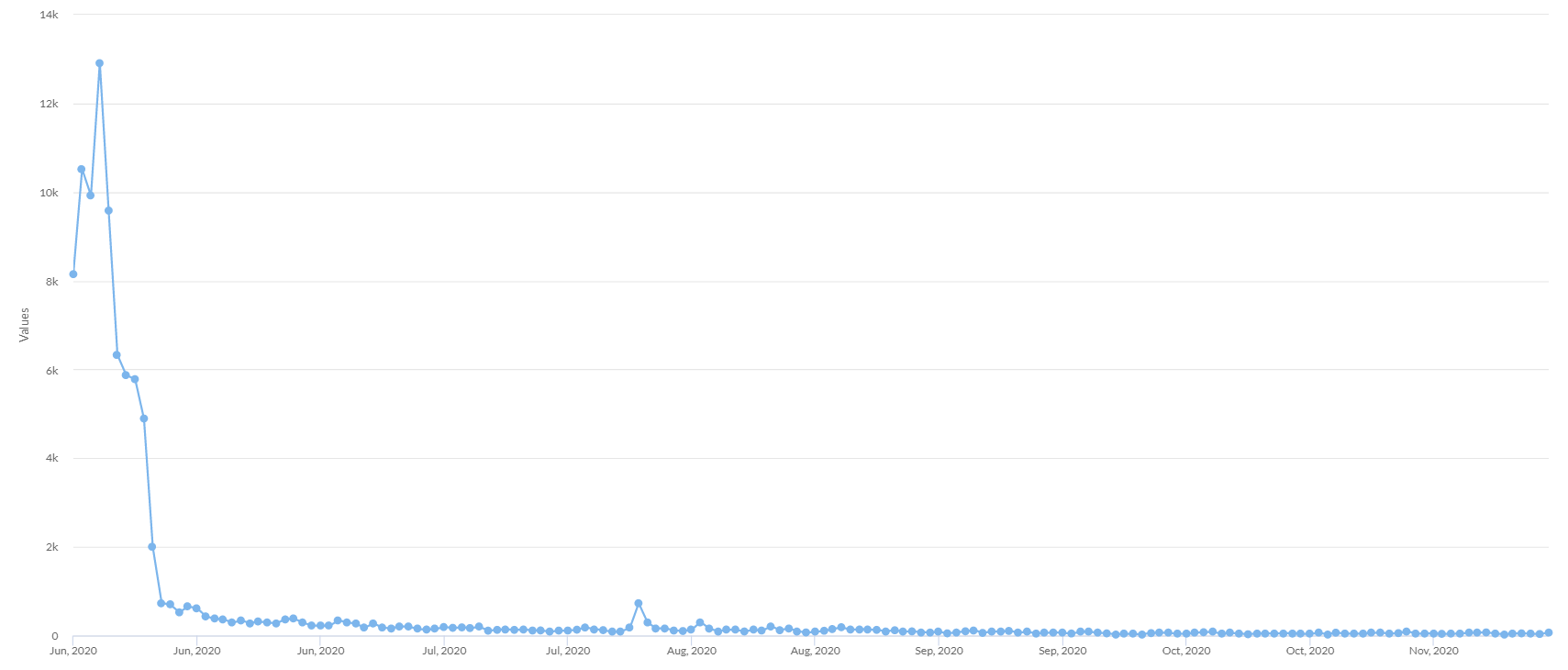
**

Time (Biweekly)

Number of Posts Including Topic Mentions

**Figure S2:** The 5 most commonly mentioned symptoms on social media during the period of June 14 to December 13, 2020.


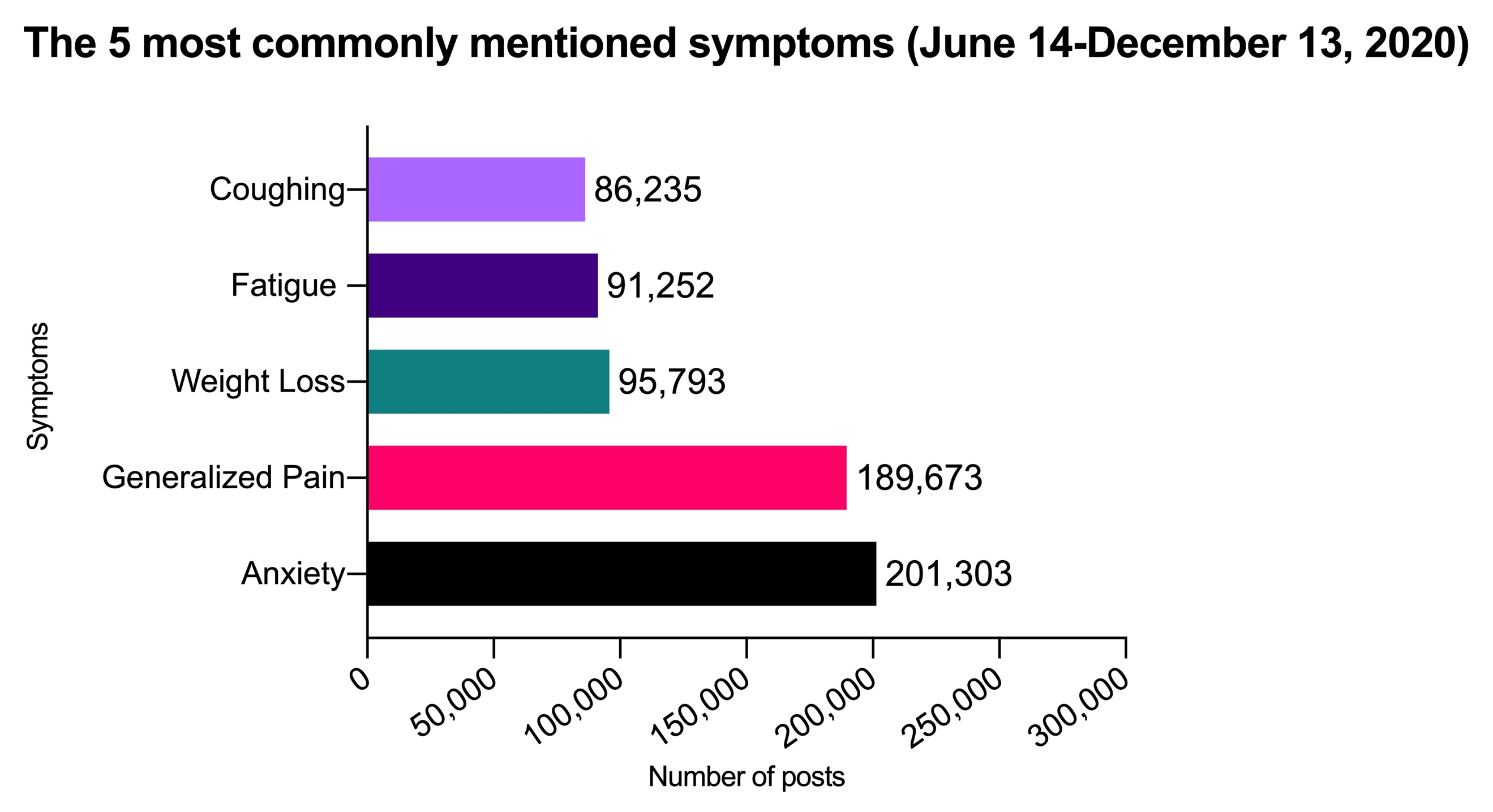


**Figure S3:** The 5 most commonly mentioned medical disorders on social media during the period of June 14 to December 13, 2020.


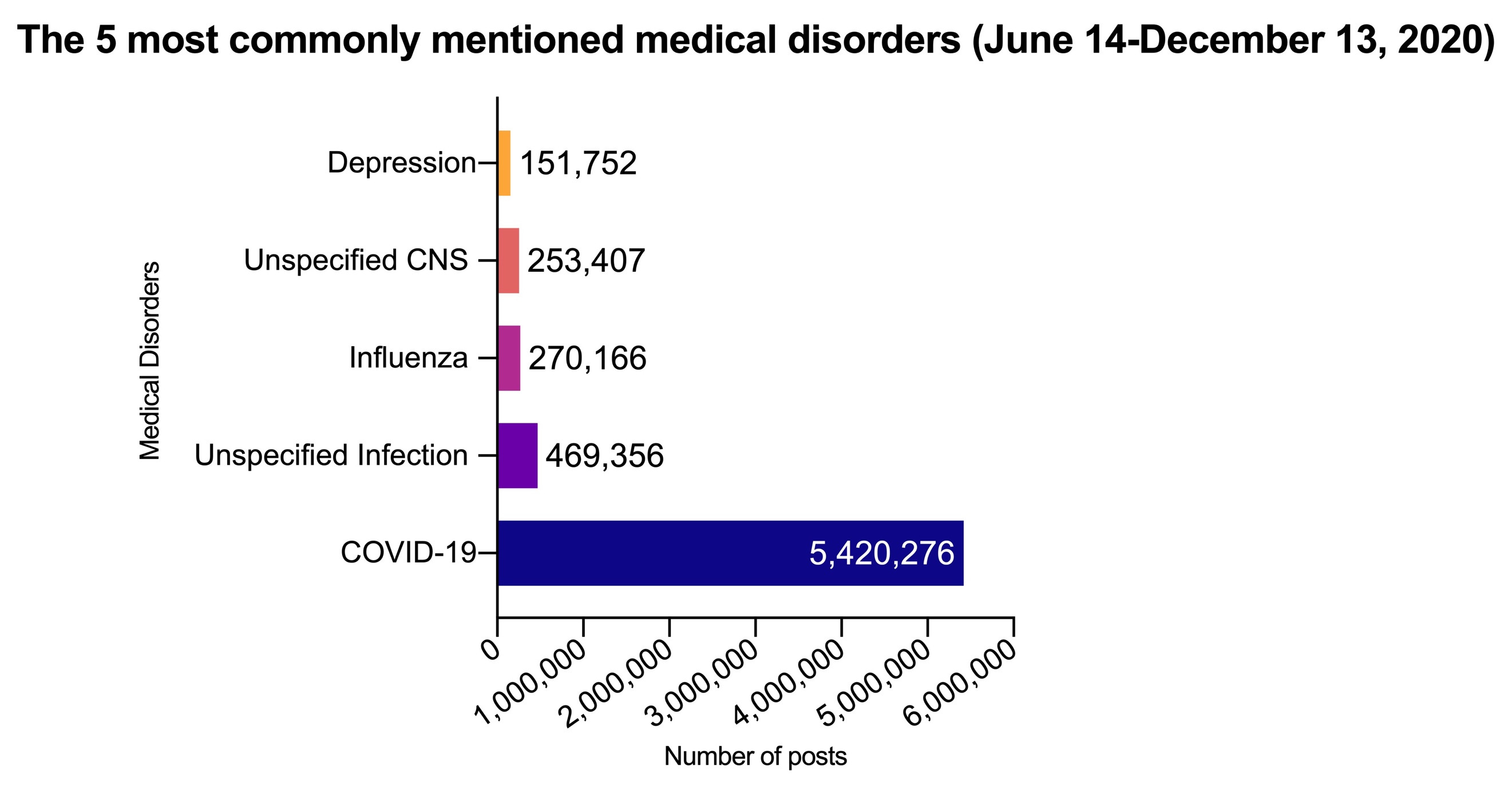


**Figure S4.** Comparing changes in the 5 most commonly mentioned symptoms on social media between June 13-Aug 31, 2020 to September 1-December 13, 2020.

**
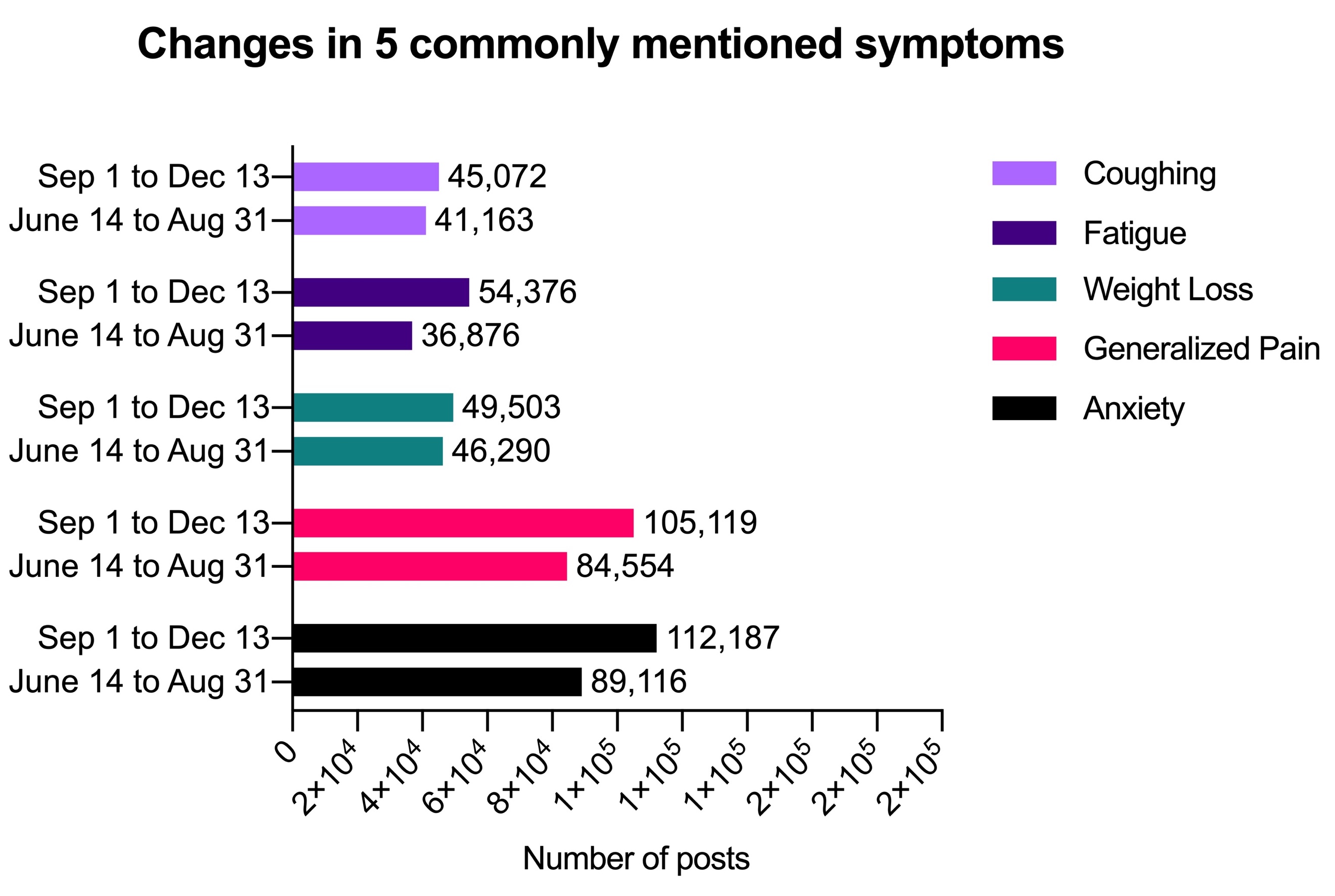
**

**Figure S5.** Comparing changes in the 5 most commonly mentioned medical disorders on social media between June 13-Aug 31, 2020 to September 1-December 13, 2020.

**
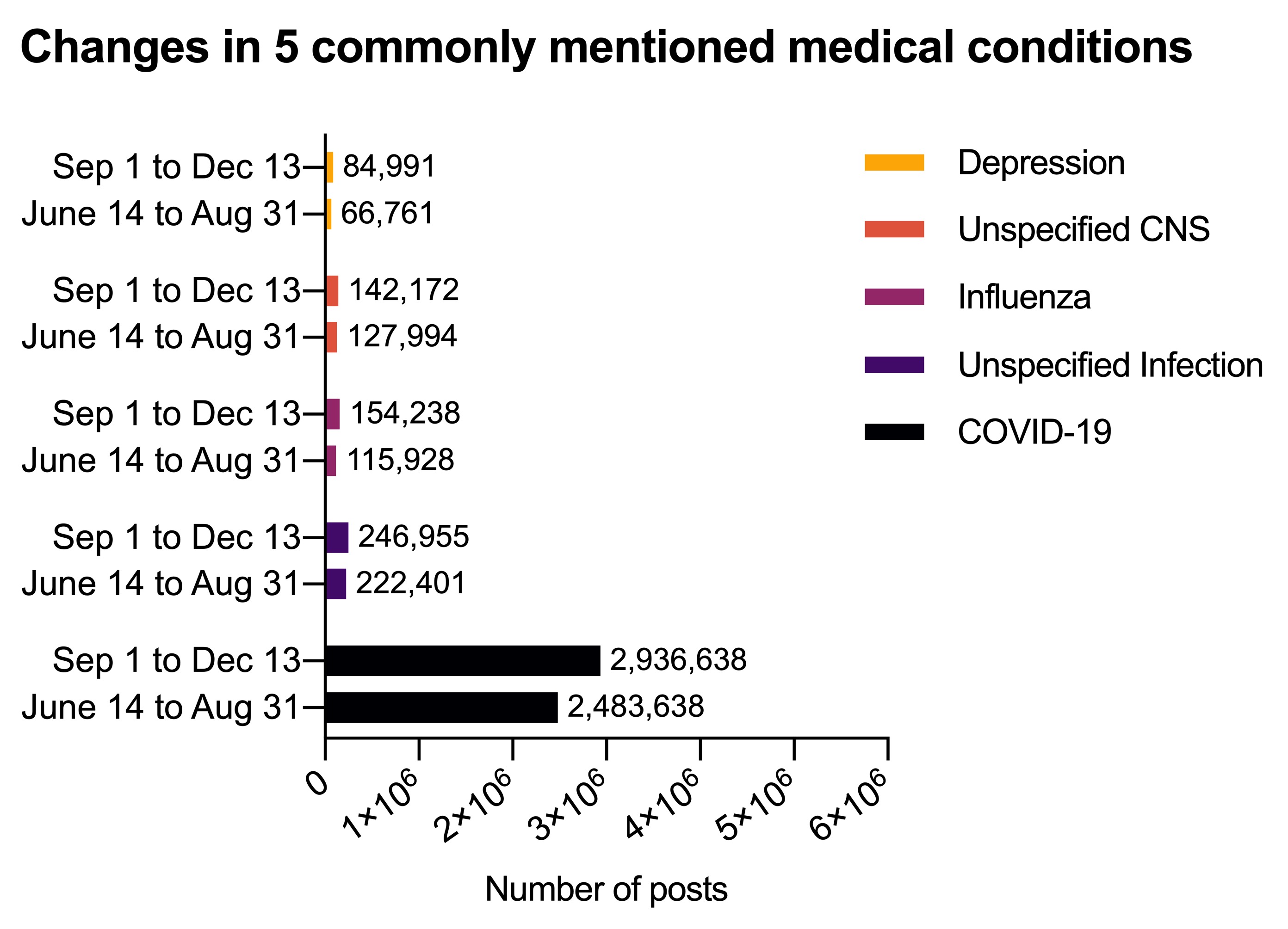
**

**Figure S6.** Comparing changes in the 10 most commonly mentioned COVID-19 symptoms between June 13-Aug 31, 2020 and September 1-December 13, 2020.


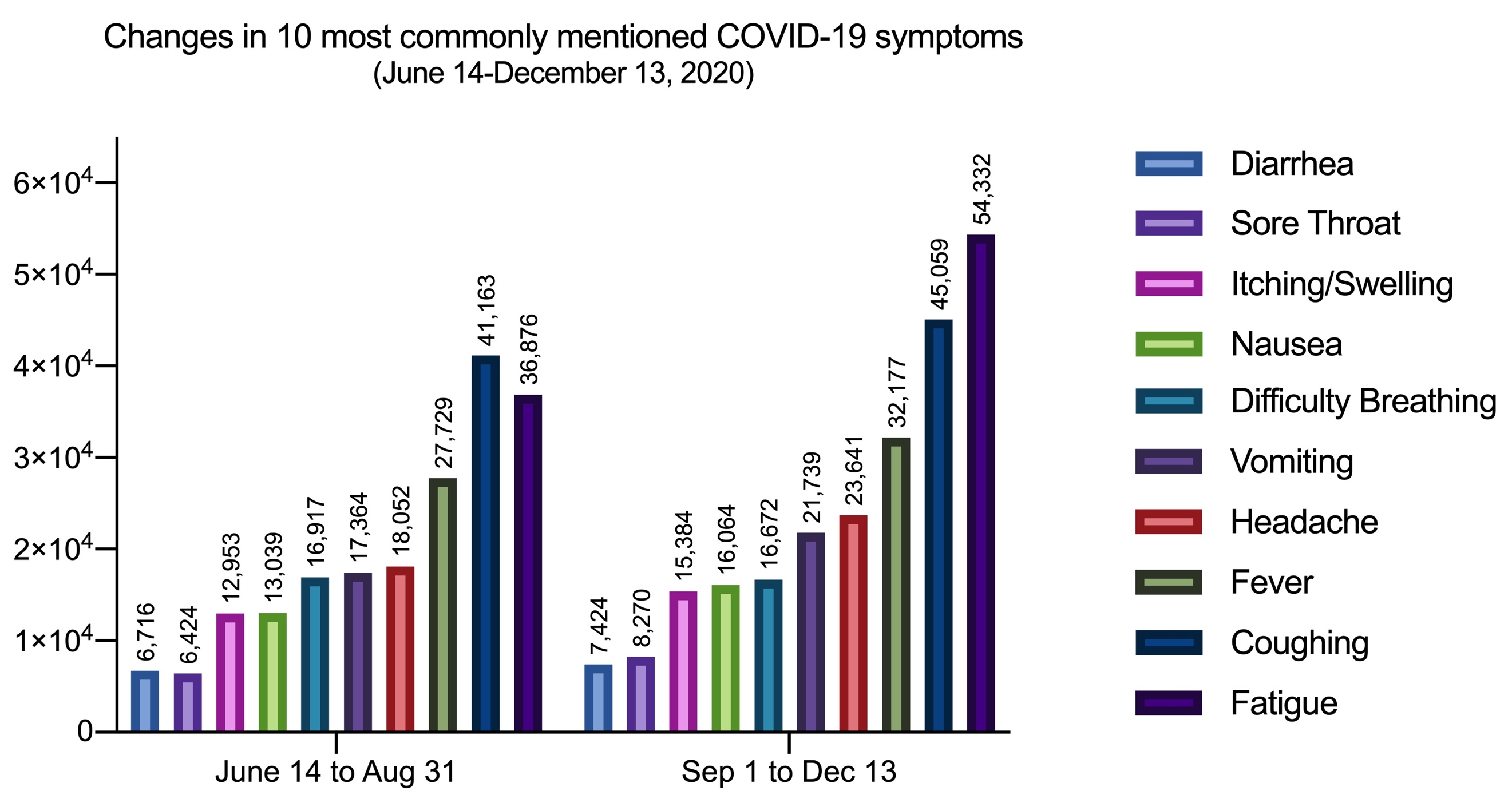


**Table S1**: Example of original posts classified as taxonomy topics by NLP algorithms.

| Original Post | Taxonomy Topic | Keywords in Text |
| --- | --- | --- |
| Hi, Rib cage pain is common after running or jogging, especially in those who started these after long time. Causes are multiple ranging from inadequate lung capacity to movement of organs. All you need to do is to go slow, start running for 10 min and then gradually increase it over a period of months and years and not over weeks or days. | **Chest Pain*** | Rib Cage *in proximity* pain |
|  | Outdoor physical activity | running; jogging |
| Wow that’s amazing!!.I’m 270lbs and feel like I have a similar body to your “before” picture. I started trying to lose weight in January and had gotten down to 250 by March, but we had to go into lockdown for 9 months due to COVID. We’ve just come out so I’m starting over again. Your progress is really inspiring, keep it up! Now I have an idea of what I’ll look like in 12 months lol. | **Weight Loss** | weight *in proximity* lose |
|  | Lockdown | Lockdown |
|  | COVID-19 | COVID |
| Yup it’s absolutely horrible. I couldn’t sleep, nightmares, rapid heartbeat, excessive sweating, depression, etc etc etc. Lasted for at least two weeks. | **Change in Sweat Production** | sweat *in proximity* excess* |
|  | Depression | Depression |
|  | Change in Heart Rate | heartbeat *in proximity* rapid |

*bold text indicated symptom and medical condition topics in our taxonomy

**Table S2**. Frequency of COVID-19 disease status and diagnostic methods discussions for the 5 most commonly mentioned symptoms and medical conditions on social media (June 14 to December 13, 2020, not indicative of a person’s COVID-19 status).

| COVID-19  Filters | **Five Most Commonly Mentioned Symptoms** | | | | |
| --- | --- | --- | --- | --- | --- |
|  | Anxiety | Generalized Pain | Weight Loss | Fatigue | Cough |
| **COVID-19 disease status**  (Percentage of posts out of total # of posts mentioning this for a specific symptom) | | | | | |
| **Tested positive** | 2,202 (37.3%) | 1,669  (37.8%) | 220  (43.7%) | 3,402 (41.4%) | 7,916  (37.3%) |
| **Tested negative** | 779  (13.2%) | 771  (17.5%) | 59  (11.7%) | 1,412 (17.2%) | 3,034  (14.3%) |
| **Symptomatic** | 1,531 (25.9%) | 742  (16.8%) | 82  (16.3%) | 1,585 (19.3%) | 4,092  (19.3%) |
| **Asymptomatic** | 869  (14.7%) | 850  (19.3%) | 79  (15.7%) | 1,257 (15.3%) | 4,920  (23.2%) |
| **Exposed to confirmed patient** | 407  (6.9%) | 227  (5.1%) | 25  (4.9%) | 350  (4.3%) | 1,038  (4.9%) |
| **Recovered** | 123  (2.1%) | 156  (3.5%) | 38  (7.6%) | 206  (2.5%) | 205  (1.0%) |
| **COVID-19 diagnostic methods**  (Percentage of posts out of total # of posts mentioning this for a specific symptom) | | | | | |
| **COVID-19 tests** | 2,981 (97.6%) | 2,440  (96.5%) | 279  (97.2%) | 4,814 (99.0%) | 10,950  (99.3%) |
| **Remotely diagnosed** | 31 (1.0%) | 45 (1.8%) | 7 (2.5%) | 27 (0.6 %) | 51 (0.5%) |
| **Self-diagnosed** | 43 (1.4%) | 43 (1.7%) | 1 (0.3%) | 21 (0.4%) | 31 (0.3%) |
| COVID-19  Filters | **Five Most Commonly Mentioned Medical Disorders** | | | | |
|  | COVID-19 | Unspecified Infection | Influenza | Unspecified CNS | Depression |
| **COVID-19 disease status** (percentage of posts out of total # of posts mentioning this for a medical disorder) | | | | | |
| **Tested positive** | 503,564 (62.9%) | 33,543 (46.5%) | 12,349 (40.4%) | 574  (38.0%) | 662  (40.8%) |
| **Tested negative** | 94,063 (11.8%) | 7,672  (10.6%) | 4,565 (14.9%) | 141  (9.3%) | 193  (11.9%) |
| **Symptomatic** | 64,065 (8.0%) | 10,073 (13.9%) | 4,657 (15.2%) | 325  (21.5%) | 331  (20.4%) |
| **Asymptomatic** | 72,985 (9.1%) | 16,008 (22.2%) | 6,944 (22.7%) | 299  (19.8%) | 214  (13.1%) |
| **Exposed to confirmed patient** | 60,208 (7.5%) | 4,413  (6.1%) | 1,883  (6.2%) | 94  (6.3%) | 134  (8.3%) |
| **Recovered** | 5,123  (0.6%) | 366  (0.5%) | 158  (0.5%) | 77  (5.1%) | 87  (5.4%) |
| **COVID-19 diagnostic methods** (percentage of posts out of total # of posts mentioning this for a specific medical disorder) | | | | | |
| **COVID-19 tests** | 597,627 (98.9%) | 41,215 (99.4%) | 16,914 (99.5%) | 715  (97.5%) | 855  (96.8%) |
| **Remotely diagnosed** | 6,408  (1.1%) | 202  (0.5%) | 48  (0.3%) | 13  (1.8%) | 9  (1.0%) |
| **Self-diagnosed** | 530  (0.1%) | 41  (0.1%) | 37  (0.2%) | 5  (0.7%) | 19  (2.2%) |

**Table S3**. Peak and trough dates of the number of social media posts for the 5 most commonly mentioned symptoms and medical conditions (June 14 and December 13, 2020).

| Classification | Peak Date | Number of posts on peak Date | Trough Date | Number of posts on trough date |
| --- | --- | --- | --- | --- |
| **The 5 most commonly mentioned symptoms** | | | | |
| Anxiety | 10/10/2020 | 1,735 | 11/23/2020 | 115 |
| Generalized pain | 10/10/2020 | 1,660 | 11/23/2020 | 143 |
| Weight loss | 7/28/2020 | 779 | 11/23/2020 | 51 |
|  | 10/10/2020 | 754 |  |  |
| Fatigue | 09/03/2020 | 730 | 11/23/2020 | 74 |
|  | 10/02/2020 | 854 |  |  |
|  | 10/10/2020 | 786 |  |  |
|  | 11/18/2020 | 782 |  |  |
| Coughing | 06/28/2020 | 1030 | 11/23/2020 | 57 |
|  | 09/08/2020 | 1212 |  |  |
|  | 10/10/2020 | 881 |  |  |
| **The 5 most commonly mentioned medical conditions** | | | | |
| COVID-19^a^ | 10/02/2020 | 73,719 | 11/23/2020 | 5113 |
| Unspecified infectious disease | 10/02/2020 | 3998 | 11/23/2020 | 330 |
| Influenza | 10/06/2020 | 3903 | 11/23/2020 | 269 |
| Unspecified CNS^b^ disorders | 10/10/2020 | 2623 | 11/23/2020 | 147 |
| Depression | 10/10/2020 | 1347 | 11/23/2020 | 113 |

Abbreviations: a, coronavirus disease 2019; b, CNS-central nervous system

**Table S4.** Daily changes in frequency of top 5 symptom posts associated with U.S. daily COVID-19 statistics during June 14 - December 13, 2020.

| Daily changes of top 5 mentioned symptoms | Daily COVID-19 statistics | Correlation,  Pearson’s r | *P-*value |
| --- | --- | --- | --- |
| Changes of anxiety | New cases | -0.49 | **0.009** |
|  | New deaths | 0.04 | 0.86 |
|  | New active cases | 0.13 | 0.50 |
|  | New recovered cases | -0.10 | 0.60 |
| Changes of generalized pain | New cases | -0.46 | **0.013** |
|  | New deaths | 0.01 | 0.97 |
|  | New active cases | 0.14 | 0.49 |
|  | New recovered cases | -0.07 | 0.74 |
| Changes of weight loss | New cases | -0.39 | **0.04** |
|  | New deaths | 0.003 | 0.99 |
|  | New active cases | 0.06 | 0.76 |
|  | New recovered cases | -0.03 | 0.89 |
| Changes of fatigue | New cases | -0.35 | 0.06 |
|  | New deaths | 0.09 | 0.63 |
|  | New active cases | 0.04 | 0.85 |
|  | New recovered cases | 0.06 | 0.76 |
| Changes of cough | New cases | -0.37 | 0.05 |
|  | New deaths | 0.03 | 0.89 |
|  | New active cases | 0.24 | 0.22 |
|  | New recovered cases | -0.08 | 0.68 |

**Table S5.** Frequency of top 5 symptom posts associated with changes in U.S. daily COVID-19 statistics during June 14 - December 13, 2020.

| Top 5 mentioned symptoms | Changes in Daily COVID-19 statistics | Correlation,  Pearson’s r | *P-*value |
| --- | --- | --- | --- |
| Anxiety | Changes of new cases | 0.02 | 0.94 |
|  | Changes of new deaths | 0.49 | **0.008** |
|  | Changes of new active cases | 0.57 | **0.002** |
|  | Changes of new recovered cases | 0.36 | 0.06 |
| Generalized pain | Changes of new cases | 0.02 | 0.93 |
|  | Changes of new deaths | 0.48 | **0.010** |
|  | Changes of new active cases | 0.59 | **0.001** |
|  | Changes of new recovered cases | 0.29 | 0.12 |
| Weight loss | Changes of new cases | -0.06 | 0.76 |
|  | Changes of new deaths | 0.39 | **0.04** |
|  | Changes of new active cases | 0.48 | **0.01** |
|  | Changes of new recovered cases | 0.31 | 0.10 |
| Fatigue | Changes of new cases | 0.09 | 0.63 |
|  | Changes of new deaths | 0.48 | **0.01** |
|  | Changes of new active cases | 0.53 | **0.004** |
|  | Changes of new recovered cases | 0.37 | **0.049** |
| Coughing | Changes of new cases | -0.09 | 0.66 |
|  | Changes of new deaths | 0.33 | 0.09 |
|  | Changes of new active cases | 0.37 | 0.06 |
|  | Changes of new recovered cases | 0.32 | 0.09 |

**Table S6.** Daily changes in frequency of top 5 symptom posts associated with changes in U.S. daily COVID-19 statistics during June 14 - December 13, 2020.

| Changes in top 5 mentioned symptoms | Changes in Daily COVID-19 statistics | Correlation,  Pearson’s r | *P-*value |
| --- | --- | --- | --- |
| Changes of anxiety | Changes of new cases | 0.13 | 0.50 |
|  | Changes of new deaths | 0.16 | 0.42 |
|  | Changes of new active cases | -0.21 | 0.29 |
|  | Changes of new recovered cases | 0.33 | 0.09 |
| Changes of generalized pain | Changes of new cases | 0.11 | 0.59 |
|  | Changes of new deaths | 0.12 | 0.54 |
|  | Changes of new active cases | -0.27 | 0.17 |
|  | Changes of new recovered cases | 0.38 | 0. 048 |
| Changes of weight loss | Changes of new cases | -0.06 | 0.76 |
|  | Changes of new deaths | 0.19 | 0.33 |
|  | Changes of new active cases | -0.27 | 0.18 |
|  | Changes of new recovered cases | 0.29 | 0.15 |
| Changes of fatigue | Changes of new cases | -0.29 | 0.14 |
|  | Changes of new deaths | 0.09 | **0.001** |
|  | Changes of new active cases | 0.48 | **0.009** |
|  | Changes of new recovered cases | 0.51 | **0.005** |
| Changes of cough | Changes of new cases | 0.10 | 0.61 |
|  | Changes of new deaths | 0.23 | 0.26 |
|  | Changes of new active cases | -0.09 | 0.65 |
|  | Changes of new recovered cases | 0.16 | 0.44 |

**Table S7.** Frequency of top 5 symptom posts associated with U.S. daily COVID-19 statistics during June 14 - December 13, 2020.

| Top 5 mentioned symptoms | Daily COVID-19 statistics | Correlation,  Pearson’s r | *P-*value |
| --- | --- | --- | --- |
| Anxiety | New cases | -0.04 | .85 |
|  | New deaths | 0.08 | .67 |
|  | New active cases | 0.05 | .78 |
|  | New recovered cases | -0.43 | **.02** |
| Generalized pain | New cases | -0.05 | .79 |
|  | New deaths | 0.06 | .76 |
|  | New active cases | 0.03 | .86 |
|  | New recovered cases | -0.44 | **.02** |
| Weight loss | New cases | -0.26 | .18 |
|  | New deaths | 0.001 | .99 |
|  | New active cases | 0 | .99 |
|  | New recovered cases | -0.55 | **.001** |
| Fatigue | New cases | 0.13 | .51 |
|  | New deaths | 0.09 | .64 |
|  | New active cases | 0.13 | .51 |
|  | New recovered cases | -0.28 | .14 |
| Cough | New cases | -0.13 | .47 |
|  | New deaths | -0.10 | .61 |
|  | New active cases | -0.06 | .76 |
|  | New recovered cases | -0.51 | **.005** |
